# Challenges and solutions to estimating tuberculosis disease incidence by country of birth in Los Angeles County

**DOI:** 10.1371/journal.pone.0209051

**Published:** 2018-12-18

**Authors:** Adam Readhead, Alicia H. Chang, Jo Kay Ghosh, Frank Sorvillo, Roger Detels, Julie Higashi

**Affiliations:** 1 Department of Epidemiology, Fielding School of Public Health, University of California Los Angeles, Los Angeles, California, United States of America; 2 TB Control Program, Los Angeles County Department of Public Health, Los Angeles, California, United States of America; 3 Independent Researcher, Los Angeles, California, United States of America; University of Washington, UNITED STATES

## Abstract

**Background:**

Among U.S. residents, tuberculosis (TB) disease disproportionally affects non-U.S.-born persons and varies substantially by country of birth. Yet TB disease incidence rates by country of birth are not routinely reported despite these large, known health disparities. This is in part due to the technical challenges of using standard regression analysis with a communicable disease. Here, we estimate tuberculosis disease incidence rates by country of birth and demonstrate methods for overcoming these challenges using TB surveillance data from Los Angeles County which has more than 3.5 million non-U.S.-born residents.

**Methods:**

Cross-sectional data on 5,447 cases of TB disease from Los Angeles County were combined with population estimates from the American Community Survey to calculate TB disease incidence rates for 2005 through 2011. Adjusted incidence rates were modelled using Poisson and negative binomial regressions. Bayesian models were used to account for the uncertainty in population estimates.

**Results:**

The unadjusted incidence rate among non-U.S.-born persons was 15 per 100,000 person-years in contrast to the rate among U.S-born persons, 2 per 100,000. The unadjusted incidence rates were 44 and 12 per 100,000 person-years among persons born in the Philippines and Mexico, respectively. In adjusted analysis, persons born in the Philippines were 2.6 (95% CI: 2.3–3.1) times as likely to be reported as a TB case than persons born in Mexico. Bayesian models showed similar results.

**Conclusion:**

This study confirms substantial disparities in TB disease by country of birth in Los Angeles County. Accounting for age, gender, years in residence and year of diagnosis, persons from the Philippines, Vietnam and several other countries had much higher rates of reported TB disease than other foreign countries. We demonstrated that incidence rates by country of birth can be estimated using available data despite technical challenges.

## Introduction

Tuberculosis (TB) is a global public health threat with more than one-quarter of the world’s population infected with *Mycobacterium tuberculosis* and, in 2016, more than 10.4 million incident cases [1, 2]. In the U.S., there were 9,272 reported TB cases in 2016, of which 69% were among non-U.S.-born persons [3]. Estimation of TB incidence by country of birth is especially important in Los Angeles County which is home to 3.5 million who were born outside the U.S.; the largest number of non-U.S.-born persons within a single U.S. county [4, 5]. Earlier studies have shown substantial disparities in TB disease incidence by country of birth both in Los Angeles County and domestically [6, 7]. Only by describing health disparities can we achieve the Federal Department of Health and Human Services’ goal of eliminating them [8].

Yet these analyses did not produce incidence rates adjusted for important confounders such as age, gender and years in residence, in part due to the technical challenges of using standard regression analysis with a communicable disease [9]. Standard count models assume that outcome events are independent, however communicable disease events are by definition dependent. Each case occurs because they were infected by another case. When using count models with communicable disease, additional attention is needed to ensure that the violation of the independence assumption does not undermine the validity of the model. Furthermore, because population estimates used in calculating incidence are commonly derived from a survey, survey error needs to be accounted for. Finally, the demographics of Los Angeles County have changed since the last study of TB incidence by country of birth in Los Angeles in 1996 [7]. The proportion of non-U.S.-born persons increased slightly from 33% in 1990 to 36% in 2000 and remained stable at 36% in 2010 [10–12]. The median age increased from 30.6 in 1990 to 32.0 in 2000 and continued to increase to 34.8 in 2010 [10, 13–15]. Here we describe unadjusted and adjusted TB disease incidence rates by country of birth in Los Angeles County and demonstrate incidence rate estimation accounting for survey error in the population estimates used and potential violations of the independence assumption that occur when standard count models are used with a communicable disease.

## Materials and methods

### Data sources

Data on 5,447 reported verified cases of tuberculosis reported between January 1, 2005 through December 31, 2011 were drawn from the tuberculosis surveillance database of the Los Angeles County Department of Public Health Tuberculosis Control Program (TBCP). California medical providers are required by state law to report persons with suspected or confirmed active TB disease and provide demographic and clinical information for these patients to the local health jurisdiction [16, 17]. TBCP staff verify the case by reviewing laboratory, microbiology, and radiographic results or other clinical information, and may request additional diagnostics. This study was deemed exempt by the Los Angeles County Department of Public Health Institutional Review Board. Tuberculosis case data contain sensitive personal health information that is protected by the federal HIPAA Privacy Rule and cannot be publicly shared. Application for access should be addressed to the Los Angeles County Department of Public Health TB Control Program.

Data used to create stratified population estimates were obtained from the Public Use Microdata Survey (PUMS), a subsample of American Community Survey (ACS) data, designed to allow custom stratifications otherwise unavailable in U.S. Census reports [18, 19]. Population estimates by country of birth are also available in table B05006 from the U.S. Census which can be accessed through American Factfinder S1 File [20]. The ACS used two sampling frames: housing units and group quarters. Housing unit addresses were sampled from the Census Bureau’s master address file and the number of persons living in each unit was ascertained; survey weights were derived from the sampling probabilities [21]. Sampling for group quarters was similar [21]. Population estimates were calculated using survey weights and confidence intervals for these estimates were calculated using replicate weights [22]. Detailed county-level numerator data were derived by aggregating data from sub-county areas called Public Use Microdata Areas (PUMAs) which are defined by PUMS. PUMS began publishing data at the PUMA level in 2005, which meant that detailed numerator data at the county level was only available starting in 2005. PUMAs are redrawn with every census and the methodology for PUMA changed substantially with the 2010 Census. Because the 2010 Census results were delayed, PUMAs were defined consistently from 2000–2011. The analysis end date of 2011 was decided on to avoid issues stemming from changes in PUMA definitions.

### Inclusions and exclusions

Beginning in 2006, the ACS sampling frame included institutional and non-institutional group quarters as defined by the U.S. Census Bureau. This group quarters definition includes Federal and state correctional facilities, local jails, homeless shelters and long-term care facilities among others. In 2005 and prior years, group quarters were not included [23]. To ensure that the numerator data matched this change in denominator data, we made the following exclusions: cases residing in correctional facilities or long-term care were excluded if they were reported in 2005 but were included if they were reported between 2006 and 2011; cases reported as homeless were excluded except for those reported between 2006 and 2011 who were living in a homeless shelter. The ACS only enumerated homeless persons who live in homeless shelters, not those living on the street or in encampments. We excluded a total of 494 (9%) cases in the following categories: 277 cases that were homeless and not housed in homeless shelters (group quarters), 49 cases reported in 2005 that were living in group quarters, 112 cases that were non-U.S.-born and missing date of arrival in the U.S., 26 cases with missing country of birth, 15 cases with countries of birth not enumerated by ACS in the study period, 14 cases diagnosed in other counties but transferred to Los Angeles County for follow-up, and 1 case that could not be assigned to a PUMA. A total of 4,953 cases were available for unadjusted analysis. To accommodate the inclusion of years in residence in the U.S. in multivariable models, the data were further limited to non-U.S.-born cases, leaving 3,945 cases for adjusted analyses. Years in residence was found to be strongly associated with TB incidence in previous studies [6]. As age was included in most models, we opted not to substitute age for years in residence for U.S.-born cases as this would introduce collinearity into the regression. Cases residing in the cities of Long Beach or Pasadena were not included as those cases are not reported to the Los Angeles County Department of Public Health.

### Data definitions

Data on cases born in South Korea and North Korea were combined into a single category, Korea, to match the ACS. Age was defined as age at diagnosis. Years in residence was defined as the difference in years between the arrival date and the diagnosis date. Report date was defined as the date the case was confirmed. Isoniazid mono-resistance was defined as resistance to isoniazid only; multi-drug resistance (MDR) was defined as resistance to isoniazid and rifampicin with or without resistance to additional TB medications. Extensively drug-resistant tuberculosis (XDR) was defined as meeting the MDR definition as well as resistance to “any fluoroquinolone and at least one of three injectable second-line drugs.” [24, 25]. Culture positive was defined as a positive culture from sputum collected within 15 calendar days of the start of treatment (if treatment was reported) or within 15 calendar days of diagnosis (if treatment was not reported) [26]. The reference category for country of birth was set as Mexico.

### Analysis

Using a cross-sectional design for the period 2005–2011, we calculated unadjusted incidence rates and standard confidence intervals using the number of TB cases and the estimated population stratified by country of birth. Confidence intervals for unadjusted rates likely understate the variability of the estimate because data were over-dispersed. In a Poisson model, the mean and variance are equal; over-dispersion is defined as when the variance is larger than the mean [27]. Relative standard error was calculated as $\frac{1}{\sqrt{n}}$ where *n* is the number of cases. We fit generalized linear models (GLMs) based on Poisson and negative binomial distributions, acknowledging that data with correlated outcomes, such as TB, are commonly over-dispersed. We monitored over-dispersion in our models via the dispersion statistic. We selected country of birth, age, gender, years in residence and year of report as covariates *a priori* based on evidence in previous literature and on availability in TB surveillance and PUMS data [6, 7, 28]. Robust confidence intervals were calculated per Hilbe [9].

To account for uncertainty in population estimates, we adopted a Bayesian framework and introduced probability distributions, or priors, for these estimates. First, we constructed Bayesian analogues to Poisson and negative binomial GLMs. Bayesian models were fitted using R, OpenBUGS, and nimble [29–31]. Standard BUGS coding was used S2 File. Priors for the intercept and all covariate coefficients were defined to be *N*(0,1000). All covariates were categorical and had corner constraints on the reference category. For the negative binomial Bayesian model, the prior for r was *G*(1,10). Two MCMC chains were run for 100,000 iterations each. Reasonable mixing and stability were achieved. Second, we introduced informative priors on the population estimates, constructed to match the standard errors of these estimates calculated using replicate weights. The population estimate priors were truncated to [1, ∞]. This resulted in a distortion of the prior distribution for some estimates. Analysis was done using SAS version 9.1.3, R version 3.4, R Studio version 1.0.143 and a variety of R packages S2 File. Bayesian models were run in OpenBUGS version 3.2.2 rev 1012 [30].

## Results

### Unadjusted analysis

The TB disease incidence rate was nearly seven times higher among non-US-born persons (15.8 per 100,000 person-years) than US-born persons (2.3 per 100,000 person-years). However, among non-US-born persons, there was considerable variation by country of birth. The incidence rate was highest among persons born in Burma (78.9 per 100,000), Ethiopia (55.9 per 100,000 person-years), Indonesia (47.4 per 100,000 person-years), the Philippines (44.3 per 100,000) and Vietnam (38.7 per 100,000) Table 1.

**Table 1:** Unadjusted TB incidence rates by selected country of birth^1^, Los Angeles County 2005^2^−2011.

Country of Birth	Cases	Person-Years^2^	Unadjusted Incidence per 100,000 person-years	95% Confidence Interval^4^	Proportion of Cases	Culture Positive	Isoniazid Resistant^5^		Multidrug Resistant^5^	
							N	%	N	%
Burma (Myanmar)	34	43,072	78.9	(55.5–109.1)	0.7	22	*	*	*	*
Ethiopia	25	44,750	55.9	(37.0–81.3)	0.5	15	*	*	*	*
Indonesia	49	103,480	47.4	(35.4–62.1)	1.0	34	*	*	*	*
Philippines	742	1,674,344	44.3	(41.2–47.6)	15.0	447	90	20.1	13	2.9
Vietnam	265	685,320	38.7	(34.2–43.5)	5.3	168	32	19	*	*
India	98	331,396	29.6	(24.1–35.9)	2.0	32	6	18.8	*	*
Nigeria	16	56,307	28.4	(16.8–45.2)	0.3	6	*	*	*	*
China	261	942,822	27.7	(24.5–31.2)	5.3	177	11	6.2	6	3.4
Cambodia (Kampuchea)	43	173,988	24.7	(18.1–33.0)	0.9	25	*	*	*	*
Pakistan	12	50,634	23.7	(12.8–40.3)	0.2	5	*	*	*	*
Peru	46	195,596	23.5	(17.4–31.1)	0.9	30	*	*	*	*
Korea	249	1,123,255	22.2	(19.5–25.1)	5.0	180	22	12.2	8	4.4
Honduras	51	234,421	21.8	(16.4–28.4)	1.0	39	*	*	*	*
Thailand	29	155,805	18.6	(12.7–26.4)	0.6	18	*	*	*	*
Guatemala	212	1,207,414	17.6	(15.3–20.0)	4.3	145	12	8.3	*	*
Mexico	1,271	10,212,974	12.4	(11.8–13.1)	25.7	750	63	8.4	5	0.7
Taiwan	55	476,938	11.5	(8.8–14.9)	1.1	40	*	*	*	*
Colombia	12	111,087	10.8	(5.9–18.4)	0.2	6	*	*	*	*
El Salvador	173	1,836,839	9.4	(8.1–10.9)	3.5	101	*	*	*	*
Armenia	40	429,402	9.3	(5.7–14.4)	0.8	30	*	*	*	*
Hong Kong	18	193,797	9.3	(6.7–12.6)	0.4	10	*	*	*	*
Nicaragua	15	210,585	7.1	(4.1–11.5)	0.3	11	*	*	*	*
Iran	49	746,784	6.6	(4.2–9.9)	1.0	31	*	*	*	*
Japan	21	317,750	6.6	(4.9–8.6)	0.4	15	*	*	*	*
United States	1,002	43,849,895	2.3	(2.1–2.4)	20.2	464	22	4.7	*	*

Source: Los Angeles County Department of Public Health, TB Control Program. Isoniazid resistance is not exclusive of other resistance.

1 Excludes countries where the relative standard error of the incidence estimate is greater or equal to 30%.

2 Person-years were calculated using the ACS Public Use Microdata Survey.

3 For 2005, we excluded cases indicated to be homeless, incarcerated or in long-term care facilities because ACS did not estimate these populations.

4 Confidence intervals were calculated using the mid-p method and are likely to understate the variability of the estimate because data were over-dispersed.

5 The number of culture positive case was used as the denominator for proportions with drug resistance.

* Data in strata with 5 or fewer cases are suppressed.

Of cases reported in the study period, 64% occurred among persons born in eight countries: the Philippines, Vietnam, China, India, Korea, Guatemala, Mexico and El Salvador. Among these eight countries, TB incidence rates ranged from 44.3 per 100,000 person-years (95% CI 41.1–47.5) among persons born in the Philippines to 9.4 per 100,000 (95% CI 8.0–10.8) among persons born in El Salvador.

The proportion of culture positive cases that were isoniazid mono-resistant was highest among persons born in the Philippines, Vietnam and India Table 1. The proportion of isoniazid resistance ranged from 18% to 20% for active TB cases born in the Philippines, Vietnam or India. In contrast, the proportion of isoniazid resistance ranged from 3% to 8% for TB cases born in Honduras, El Salvador or Mexico. Similar patterns were observed for multi-drug resistant (MDR) cases, though very few countries of birth could be analyzed due to sparse data. Of cases among persons born in Mexico, 0.7% were MDR, whereas 4.4% of cases among persons born in Korea were MDR. There were no cases with extensively-drug resistant TB in the analysis period.

Among non-U.S.-born persons, TB disease incidence rates were higher among men and those residing in the U.S. for less than 1 year; TB disease increased with age Table 2 and S1 Table. Of all non-U.S.-born cases in the study period, 59% were among persons who had resided in the U.S. for 10 or more years. The overall incidence rate declined from 18.1 per 100,000 in 2005 to 14.0 per 100,000 in 2011. Among the top 8 countries of birth with highest burden of TB disease, incidence rates declined though rates were variable S2 Table.

**Table 2:** Unadjusted and adjusted TB incidence rates and incidence rate ratios by selected country of birth, Los Angeles County 2005^1^−2011.

		Unadjusted			Adjusted Poisson GLM			Adjusted negative binomial GLM		
Demographic Characteristic		Incidence	Incidence Rate Ratio	Standard 95% CI	Incidence Rate Ratio	Standard 95% CI	Robust 95% CI	Incidence Rate Ratio	Standard 95% CI	Robust 95% CI
Country of Birth										
	Philippines	44.3	3.56	3.25–3.90	2.56	2.33–2.81	2.29–2.86	2.60	2.31–2.94	2.31–2.93
	Vietnam	38.5	3.10	2.71–3.53	2.60	2.27–2.99	2.26–2.99	2.66	2.26–3.13	2.30–3.08
	India	29.6	2.38	1.93–2.92	1.47	1.19–1.82	1.16–1.87	1.51	1.20–1.89	1.19–1.92
	China	27.7	2.22	1.95–2.54	1.27	1.10–1.45	1.08–1.49	1.26	1.08–1.48	1.07–1.49
	Korea	22.2	1.78	1.56–2.04	1.35	1.18–1.55	1.15–1.57	1.36	1.16–1.59	1.15–1.59
	Guatemala	17.6	1.41	1.22–1.63	1.27	1.09–1.47	1.06–1.52	1.27	1.07–1.51	1.05–1.53
	Mexico	12.4	Reference		Reference			Reference		
	Other non-U.S. country	9.6	0.77	0.70–0.85	0.59	0.54–0.65	0.53–0.67	0.63	0.55–0.71	0.56–0.70
	El Salvador	9.4	0.76	0.65–0.89	0.75	0.64–0.88	0.63–0.88	0.75	0.63–0.91	0.64–0.89
Age at Diagnosis (years)										
	0–19	6.6	0.46	0.38–0.56	0.19	0.16–0.24	0.16–0.24	0.20	0.16–0.25	0.16–0.25
	20–39	12.1	0.85	0.78–0.92	0.58	0.53–0.63	0.52–0.64	0.61	0.55–0.68	0.55–0.68
	40–59	14.2	Reference		Reference			Reference		
	60–79	25.8	1.81	1.67–1.97	1.87	1.72–2.04	1.70–2.06	1.91	1.72–2.13	1.72–2.13
	80–106	49.6	3.49	3.12–3.90	4.13	3.68–4.64	3.65–4.67	4.20	3.66–4.82	3.69–4.78
Gender										
	Male	18.7	Reference		Reference			Reference		
	Female	12.9	0.69	0.65–0.73	0.63	0.59–0.67	0.58–0.68	0.65	0.60–0.70	0.60–0.70
Years in Residence										
	0–1	109.2	9.22	8.19–10.39	12.4	10.87–14.19	10.48–14.71	12.8	11.05–14.83	10.83–15.14
	2–4	31.2	2.64	2.42–2.87	4.35	3.96–4.77	3.92–4.81	4.33	3.87–4.84	3.90–4.81
	5–9	17.5	1.47	1.34–1.62	2.30	2.09–2.54	2.06–2.58	2.31	2.06–2.60	2.07–2.59
	10–19	13.9	1.17	1.06–1.30	1.65	1.48–1.84	1.46–1.87	1.72	1.52–1.94	1.51–1.95
	20–93	11.8	Reference		Reference			Reference		
Year of Diagnosis										
	2005	18.1	Reference		Reference			Reference		
	2006	17.8	0.98	0.88–1.09	1.00	0.90–1.12	0.89–1.13	1.00	0.87–1.15	0.88–1.14
	2007	16.4	0.90	0.81–1.01	0.89	0.79–1.00	0.78–1.02	0.90	0.78–1.03	0.78–1.04
	2008	15.7	0.86	0.77–0.97	0.86	0.77–0.97	0.75–0.99	0.86	0.74–0.99	0.74–0.98
	2010	14.3	0.79	0.70–0.88	0.79	0.70–0.89	0.69–0.90	0.77	0.66–0.89	0.66–0.89
	2009	14.0	0.77	0.69–0.87	0.77	0.69–0.87	0.68–0.89	0.75	0.65–0.86	0.65–0.86
	2011	14.0	0.77	0.69–0.87	0.78	0.69–0.87	0.68–0.89	0.75	0.65–0.87	0.65–0.87
Dispersion Statistic		N/A			1.5			1.4		
AIC		N/A			6560			6502		

Source: Los Angeles County Department of Public Health, TB Control Program & Public Use Microdata Survey via IPUMS.

1 For 2005, we excluded cases indicated to be homeless, incarcerated or in long-term care facilities because ACS did not estimate these populations.

2 Adjusted model includes the following covariates: age, gender, length of residence and year of diagnosis.

### Adjusted analysis

Fitting a naïve Poisson GLM confirmed substantial over-dispersion; a Poisson model with country of birth and offset alone had a dispersion statistic of 10.5. An expanded model including country of birth, age, gender, report year and years in residence had notably less over-dispersion with a dispersion statistic of 1.5.

Using standard GLMs and adjusting for other factors associated with TB disease incidence such as age, gender, length of residence and report year, persons born in Vietnam were 2.6 (95% robust confidence interval (RCI) 2.3–3.0) times as likely to have been reported with TB disease than persons born in Mexico Table 2. Persons born in the Philippines and India were 2.6 (95% RCI 2.3–2.8) and 1.5 (95% RCI 1.2–1.8) times as likely, respectively, to be reported with TB disease than persons born in Mexico. In contrast, persons born in El Salvador were 0.8 (95% RCI0.6–0.9) times as likely as persons born in Mexico to be reported with TB disease. Estimates from adjusted Poisson and negative binomial models were similar and had relatively little over-dispersion Table 2. Both had dispersion statistics close to 1, though the negative binomial model was a better fit for the data.

We found negligible differences between the standard GLMs and their Bayesian analogues, though incidence rates estimated using the Bayesian negative binomial model were generally lower than standard negative binomial regression results Tables 2 and 3. Results from the Bayesian Negative Binomial model with priors on population estimates were on par with the Bayes Negative Binomial model which did not account for uncertainty in population estimates Table 3.

**Table 3:** TB incidence rate ratios by selected demographic characteristics–adjusted Bayesian models, Los Angeles County 2005^1^−2011.

Demographic Characteristic		Poisson Bayes	Negative Binomial Bayes	Negative Binomial Bayes with priors on population estimates^2^
Country of Birth				
	Philippines	2.56 (2.32–2.82)	2.60 (2.27–2.95)	2.61 (2.30–2.95)
	Vietnam	2.60 (2.24–2.98)	2.65 (2.21–3.13)	2.64 (2.21–3.12)
	India	1.47 (1.17–1.80)	1.50 (1.16–1.89)	1.50 (1.16–1.89)
	China	1.26 (1.09–1.45)	1.26 (1.06–1.48)	1.26 (1.06–1.48)
	Korea	1.34 (1.16–1.54)	1.35 (1.13–1.59)	1.36 (1.15–1.60)
	Guatemala	1.26 (1.08–1.46)	1.27 (1.05–1.51)	1.25 (1.05–1.49)
	Mexico	Reference	Reference	Reference
	Other non-U.S. country	0.59 (0.54–0.65)	0.63 (0.55–0.71)	0.62 (0.55–0.71)
	El Salvador	0.74 (0.63–0.87)	0.75 (0.61–0.90)	0.75 (0.61–0.90)
Age				
	0–19	0.19 (0.16–0.24)	0.20 (0.16–0.24)	0.20 (0.16–0.24)
	20–39	0.58 (0.53–0.63)	0.61 (0.55–0.68)	0.61 (0.55–0.68)
	40–59	Reference	Reference	Reference
	60–79	1.87 (1.71–2.04)	1.91 (1.71–2.14)	1.90 (1.69–2.11)
	80–106	4.13 (3.65–4.64)	4.20 (3.62–4.83)	4.16 (3.60–4.79)
Gender				
	Male	Reference	Reference	Reference
	Female	0.63 (0.59–0.67)	0.65 (0.60–0.70)	0.65 (0.59–0.70)
Years in Residence				
	0–1	12.39 (10.77–14.16)	12.79 (10.89–14.91)	12.22 (10.36–14.36)
	2–4	4.34 (3.94–4.77)	4.32 (3.83–4.86)	4.37 (3.87–4.90)
	5–9	2.30 (2.08–2.54)	2.31 (2.03–2.61)	2.31 (2.04–2.60)
	10–19	1.65 (1.48–1.84)	1.71 (1.49–1.95)	1.69 (1.48–1.92)
	20–93	Reference	Reference	Reference
Year of Diagnosis				
	2005	Reference	Reference	Reference
	2006	1.00 (0.89–1.12)	1.00 (0.86–1.15)	0.99 (0.85–1.14)
	2007	0.89 (0.79–1.00)	0.90 (0.77–1.04)	0.89 (0.76–1.02)
	2008	0.86 (0.76–0.97)	0.85 (0.73–0.99)	0.85 (0.73–0.98)
	2009	0.79 (0.69–0.89)	0.77 (0.65–0.89)	0.77 (0.66–0.89)
	2010	0.77 (0.68–0.87)	0.75 (0.64–0.87)	0.75 (0.64–0.87)
	2011	0.77 (0.68–0.87)	0.75 (0.64–0.87)	0.75 (0.64–0.87)

Source: Los Angeles County Department of Public Health, TB Control Program & Public Use Microdata Survey via IPUMS.

1 For 2005, we excluded cases indicated to be homeless, incarcerated or in long-term care facilities because ACS did not estimate these populations.

2 Priors on population estimates calculated based on standard error of denominator which in turn was calculated based on PUMS replicate weights.

## Discussion

This study highlights the large differences in TB disease by country of birth while demonstrating solutions to common challenges in calculating unadjusted and adjusted TB incidence rates by country of birth. Earlier studies of TB disease by country of birth did not adjust for age, gender, or length of residence in the U.S., all important cofounders of TB disease risk. Here, we showed that sizable disparities by country of birth were evident even when adjusting for these confounders. In the adjusted analysis, persons born in the Philippines or Vietnam were approximately 3 times more likely than persons born in Mexico to be reported as a case of TB disease. In contrast, persons born in China, Korea or Guatemala were about 25% more likely to be reported as a case of TB disease. Overall, TB disease was more than 8 times more likely to be reported among non-U.S.-born persons than among U.S.-born persons. Previous data in Los Angeles County from 1996 showed non-U.S.-born persons to be 4 times as likely as U.S.-born persons to be diagnosed with TB in unadjusted analyses [7]. In the U.S. in 2015, non-U.S.-born persons were approximately 13 times as likely to be diagnosed with TB as U.S.-born persons [32]. Locally, this information can be used to focus population and neighborhood centered outreach for TB elimination and to ensure this outreach is implemented in a patient centered manner. Country of birth is helpful in suggesting cultural and linguistic needs of the target populations. More generally, this analysis addresses three main concerns regarding the calculation of TB disease incidence rate by country of birth. First, population estimates by country of birth can be hard to find. Here we outlined how to access population estimates by country of birth through American FactFinder and provided information on the use of PUMS data. Second, some analysts are wary of using survey estimates because of the survey error associated with these estimates. We addressed this by adding uncertainty into the model in the form of priors on the population estimates; in this case, increased uncertainty in the population estimates had little effect on the model estimates. Third, data on communicable diseases, such as TB, are typically over-dispersed and difficult to properly model with standard regression methods. Standard count models assume that outcomes are independent, but in TB, as in other communicable diseases, outcomes are not independent. However, with the inclusion of key covariates and use of appropriate diagnostic tests, these models can be used to produce reasonable estimates. Moreover, dependent outcomes typical of communicable diseases are less of a concern here because an estimated 85% of TB cases in California result from the reactivation of latent TB infection [33]. These reactivation cases are independent of each other as reactivation depends on host immune status and co-morbidities, not on recent infection.

The results also indicate a notable decline in TB disease incidence over the study period for all countries of birth S2 Table. Incidence rates declined for both non-U.S.-born persons and U.S.-born persons during the study period from 18.1 per 100,000 in 2005 to 14.0 per 100,000 in 2011; similar declines have been seen both nationally and in other cities [28, 32]. Consistent with previous reports, we observed higher incidence rates among males, older adults and, most notably, those with fewer years of residence in the U.S Table 3 [6, 7]. Additional risk among older non-U.S-born adults is likely due to longer periods of exposure in a high-burden country as well as greater likelihood of reactivation due to a waning immune system. A proxy indicator of time living in a high burden country would have been age at immigration, which we did not specifically include as a covariate. However, including both age years in US residence in models would account for age at immigration indirectly. Though we have tried to address known and suspected confounders, as with all models, residual confounding most likely remains and we acknowledge this potential limitation.

While based on mature data collection systems, this study has several limitations. Despite adjustment, the data remained over-dispersed, which could result in the under-coverage of confidence intervals, including robust confidence intervals. Confidence intervals also do not account for unknown or residual confounding or other systematic errors. If the confidence intervals presented here are too narrow, there may be little difference in the incidence rates of persons born in India, China, Korea or Guatemala and the reference category, Mexico, accounting for other covariates. Thus, the differences in TB incidence rates noted here, while in keeping with published literature, should be confirmed with additional data from other settings. Spatial effects and disease transmission were not taken into consideration. Case ascertainment is unknown though it is thought to be high, similar to other TB surveillance systems [34–36]. Higher TB disease incidence among recently-immigrated non-U.S. born could be in part the result of increased ascertainment in this group. Truncation of the population estimate priors may have inflated some population estimates, but did so minimally, given the similarity between models with and without these priors. Furthermore, some non-U.S.-born populations may be under-counted in the ACS; a recent report suggests reasonable survey coverage of non-Hispanic non-U.S.-born populations but under-coverage of Hispanic non-U.S.-born persons [37].

## Conclusions

This study confirms substantial differences in TB disease by country of birth in Los Angeles County. Even accounting for differences in age, gender, and years in residence distributions, persons from the Philippines, Vietnam and several other countries have much higher rates of reported TB disease than persons from other non-U.S. countries. We have demonstrated that incidence rates by country of birth can be calculated with readily-accessible population estimates and that more complex adjusted estimates can be achieved through the use of negative binomial models and Bayesian techniques. This analysis helped better describe the local TB disparities in Los Angeles County and can be used to inform a continued public health response.

## Supporting information

S1 File Quick guide to population estimates by country of birth. (DOCX)

S2 File OpenBUGS model code and r packages used. (DOCX)

S1 Table TB Incidence rates among Non-U.S.-born persons by demographic characteristic, los angeles county 2005–2011. (DOCX)

S2 Table TB Incidence rates among selected country of birth by year, los angeles county 2005–2011. (DOCX)
