# Supplementary material for: Challenges and solutions to estimating tuberculosis disease incidence by country of birth in Los Angeles County
Source: PLoS One. 2018 Dec 18;13(12):e0209051. doi: 10.1371/journal.pone.0209051 (PMC6298681; doi:10.1371/journal.pone.0209051)
Supplement: S1 File — (DOCX) [file pone.0209051.s001.docx]

Supplemental File 1. Quick Guide to Population Estimates by Country of Birth.

1. Navigate to American Factfinder Advanced Search, <https://factfinder.census.gov>
2. Enter “B05006” into table name box
3. Select “B05006: PLACE OF BIRTH FOR THE FOREIGN-BORN POPULATION” from available selections
4. Enter jurisdiction for which you have case counts by country of birth
5. Select the standardized jurisdiction name from selection
6. From resulting table list, select appropriate year/table
   1. For one year of case counts, choose the appropriate year and the dataset marked “ACS 1-year estimates”, e.g. “2015 ACS 1-year estimates”
   2. Choose the appropriate year and dataset marked “ACS 5-year estimates.” ACS 5 year estimates are labelled with the final year of data collection. For example, the 2015 5-year estimates represented data from 2011-2015 averaged. Equivalent to one year estimate but with smaller MOE
7. Download data
