# Supplementary material for: Challenges and solutions to estimating tuberculosis disease incidence by country of birth in Los Angeles County
Source: PLoS One. 2018 Dec 18;13(12):e0209051. doi: 10.1371/journal.pone.0209051 (PMC6298681; doi:10.1371/journal.pone.0209051)
Supplement: S2 File — (DOCX) [file pone.0209051.s002.docx]

Supplemental File 2. OpenBUGS Model Code and R Packages Used

model{

for (i in 1:numrec) {

numer[i] ~ dpois(mu[i]) #Poisson numerator declaration

# alternatively negative binomial numerator declaration:

# numer[i] ~ dnegbin(p[i], r)

# p[i] <- r/(r + mu[i])

log(mu[i]) <-

alpha +

beta.cob[cobn[i]] +

beta.age[agecatn[i]] +

beta.sex[sexn[i]] +

beta.year[yearn[i]] +

beta.yres[yres_catn[i]] +

log(denom[i])

}

alpha ~ dnorm(0,0.00001)

beta.cob[1] <- 0

for (j in 2:numcob) {

beta.cob[j] ~ dnorm(0,0.001)

}

beta.age[1] <- 0

for (k in 2:numage) {

beta.age[k] ~ dnorm(0,0.001)

}

beta.sex[1] <- 0

for (m in 2:numsex) {

beta.sex[m] ~ dnorm(0,0.001)

}

beta.year[1] <- 0

for (n in 2:numyear) {

beta.year[n] ~ dnorm(0,0.001)

}

beta.yres[1] <- 0

for (p in 2:numyres) {

beta.yres[p] ~ dnorm(0,0.001)

}

}

R packages used:

Grolemund G, Wickham H. Dates and Times Made Easy with lubridate. Journal of Statistical Software. 2011;40(3):1-25.

Hadley Wickham RF, Lionel Henry, Kirill Müller. dplyr: A Grammar of Data Manipulation. 2017.

Harrell Jr FE, contributions from Charles Dupont and many others. Hmisc: Harrell Miscellaneous. 2017.

Hilbe JM. COUNT: Functions, Data and Code for Count Data. 2016.

Matt Dowle AS. data.table: Extension of `data.frame`. 2017.

Wickham H. ggplot2: Elegant Graphics for Data Analysis: Springer-Verlag New York; 2009.

Wickham H. tidyr: Easily Tidy Data with 'spread()' and 'gather()' Functions. 2017.

Wickham KMaH. tibble: Simple Data Frames. 2017.

Lumley T. Analysis of Complex Survey Samples. Journal of Statistical Software. 2004;9(1):1-19.

Gelman SSaULaA. R2WinBUGS: A Package for Running WinBUGS from R. Journal of Statistical Software. 2005;12(3):1-16.
