# Supplementary material for: Challenges and solutions to estimating tuberculosis disease incidence by country of birth in Los Angeles County
Source: PLoS One. 2018 Dec 18;13(12):e0209051. doi: 10.1371/journal.pone.0209051 (PMC6298681; doi:10.1371/journal.pone.0209051)
Supplement: S1 Table — (DOCX) [file pone.0209051.s003.docx]

**Supplemental Table 1. TB Incidence Rates among Non-U.S.-born Persons by Demographic Characteristic, Los Angeles County 2005^1^-2011.**

| **Demographic Characteristic** | | **Cases** | **Person-Years** | **Unadjusted Incidence  per 100,000** | **95% Confidence Interval** | **Percentage of Total Cases** |
| --- | --- | --- | --- | --- | --- | --- |
| Age at Diagnosis (years) | |  |  |  |  |  |
|  | 0 – 19 | 118 | 1,787,626 | 6.6 | (5.4 - 7.8) | 3% |
|  | 20 – 39 | 1,098 | 9,085,859 | 12.1 | (11.4 - 12.8) | 28% |
|  | 40 – 59 | 1,365 | 9,597,152 | 14.2 | (13.5 - 15.0) | 35% |
|  | 60 – 79 | 976 | 3,782,404 | 25.8 | (24.2 - 27.4) | 25% |
|  | 80 – 106 | 389 | 784,359 | 49.6 | (44.7 - 54.5) | 10% |
|  |  |  |  |  |  |  |
| Gender |  |  |  |  |  |  |
|  | Male | 2,296 | 12,252,800 | 18.7 | (18.0 - 19.5) | 58% |
|  | Female | 1,650 | 12,784,600 | 12.9 | (12.3 - 13.5) | 42% |
|  |  |  |  |  |  |  |
|  |  |  |  |  |  |  |
| Years in Residence | |  |  |  |  |  |
|  | 0 – 1 | 576 | 824,925 | 69.8 | (64.1 - 75.5) | 15% |
|  | 2 – 4 | 462 | 1,774,188 | 26.0 | (23.7 - 28.4) | 12% |
|  | 5 – 9 | 579 | 3,316,024 | 17.5 | (16.0 - 18.9) | 15% |
|  | 10 – 19 | 881 | 6,823,639 | 12.9 | (12.1 - 13.8) | 22% |
|  | 20 – 93 | 1,448 | 12,298,624 | 11.8 | (11.2 - 12.4) | 37% |
|  |  |  |  |  |  |  |
| Year of Diagnosis | |  |  |  |  |  |
|  | 2005 | 652 | 3,593,316 | 18.1 | (16.8 - 19.5) | 17% |
|  | 2006 | 636 | 3,569,735 | 17.8 | (16.4 - 19.2) | 16% |
|  | 2007 | 597 | 3,642,877 | 16.4 | (15.1 - 17.7) | 15% |
|  | 2008 | 554 | 3,538,054 | 15.7 | (14.4 - 17.0) | 14% |
|  | 2009 | 501 | 3,567,900 | 14.0 | (12.8 - 15.3) | 13% |
|  | 2010 | 507 | 3,553,789 | 14.3 | (13.0 - 15.5) | 13% |
|  | 2011 | 499 | 3,571,729 | 14.0 | (12.7 - 15.2) | 13% |
|  |  |  |  |  |  |  |

1 *For 2005, we excluded cases indicated to be homeless, incarcerated or in long-term care facilities because ACS did not estimate these populations.*
