# Supplementary material for: Challenges and solutions to estimating tuberculosis disease incidence by country of birth in Los Angeles County
Source: PLoS One. 2018 Dec 18;13(12):e0209051. doi: 10.1371/journal.pone.0209051 (PMC6298681; doi:10.1371/journal.pone.0209051)
Supplement: S2 Table — (DOCX) [file pone.0209051.s004.docx]

**Supplemental Table 2. TB Incidence Rates among Selected Country of Birth by Year,**

**Los Angeles County 2005^1^-2011.**

| **Country of Birth** | **Year of Diagnosis** | | | | | | |
| --- | --- | --- | --- | --- | --- | --- | --- |
|  | 2005 | 2006 | 2007 | 2008 | 2009 | 2010 | 2011 |
| Philippines | 43.6 | 53.7 | 46.7 | 40.2 | 41.3 | 40.1 | 39.5 |
| Vietnam | 40.1 | 35.8 | 44.5 | 36.4 | 27.4 | 31.0 | 32.0 |
| India | 45.2 | * | 33.1 | 34.6 | 25.1 | * | 20.2 |
| China | 35.3 | 32.1 | 24.1 | 28.6 | 26.8 | 27.9 | 18.1 |
| Korea | 21.2 | 28.9 | 20.6 | 24.1 | 21.1 | 15.7 | 23.1 |
| Guatemala | 17.9 | 20.0 | 20.7 | 13.8 | 20.0 | 14.1 | 13.8 |
| Mexico | 15.0 | 13.9 | 11.8 | 12.4 | 11.7 | 11.2 | 10.8 |
| Other non-U.S. country | 11.4 | 11.6 | 9.9 | 9.5 | 6.8 | 9.9 | 7.9 |
| El Salvador | 10.2 | 7.1 | 10.9 | 11.7 | 6.4 | 6.4 | 12.6 |

** Incidence rates with relative standard error greater than 30% are suppressed.*

1 *For 2005, we excluded cases indicated to be homeless, incarcerated or in long-term care facilities because ACS did not estimate these populations.*
